# Using brief reflections to capture and evaluate end-user engagement: a case example using the COMPASS study

**DOI:** 10.1186/s12874-024-02222-5

**Published:** 2024-05-02

**Authors:** Princess E. Ackland, Hildi J. Hagedorn, Marie E. Kenny, Hope A. Salameh, Shannon M. Kehle-Forbes, Allison M. Gustavson, Leyla E. Karimzadeh, Laura A. Meis

**Affiliations:** 1 Center for Care Delivery and Outcomes Research, Minneapolis Veterans Affairs Health Care System, One Veterans Drive (152), Minneapolis, MN 55417 USA; 2 Department of Medicine, University of Minnesota Medical School, 420 Delaware St SE, Minneapolis, MN 55455 USA; 3 Department of Psychiatry & Behavioral Sciences, University of Minnesota Medical School, 2312 South 6th Street, Minneapolis, MN 55454 USA; 4 Center of Excellence in Substance Addiction Treatment and Education, Seattle Division, VA Puget Sound Healthcare System, 1660 S. Columbian Way, Seattle, WA 98108 USA; 5 Women’s Health Sciences Division at VA Boston, National Center for PTSD, 150 South Huntington Street, Boston, MA 02130 USA

**Keywords:** Participatory research, Qualitative methods, Effectiveness trial, Veterans, Evaluation methods

## Abstract

**Background:**

Use of participatory research methods is increasing in research trials. Once partnerships are established with end-users, there is less guidance about processes research teams can use to successfully incorporate end-user feedback. The current study describes the use of a brief reflections process to systematically examine and evaluate the impact of end-user feedback on study conduct.

**Methods:**

The Comparative Effectiveness of Trauma-Focused and Non-Trauma- Focused Treatment Strategies for PTSD among those with Co-Occurring SUD (COMPASS) study was a randomized controlled trial to determine the effectiveness of trauma-focused psychotherapy versus non-trauma-focused psychotherapy for Veterans with co-occurring posttraumatic stress disorder and substance use disorder who were entering substance use treatment within the Department of Veterans Affairs. We developed and paired a process of “brief reflections” with our end-user engagement methods as part of a supplemental evaluation of the COMPASS study engagement plan. Brief reflections were 30-minute semi-structured discussions with the COMPASS Team following meetings with three study engagement panels about feedback received regarding study issues. To evaluate the impact of panel feedback, 16 reflections were audio-recorded, transcribed, rapidly analyzed, and integrated with other study data sources.

**Results:**

Brief reflections revealed that the engagement panels made recommended changes in eight areas: enhancing recruitment; study assessment completion; creating uniformity across Study Coordinators; building Study Coordinator connection to Veteran participants; mismatch between study procedures and clinical practice; therapist skill with patients with active substance use; therapist burnout; and dissemination of study findings. Some recommendations positively impact study conduct while others had mixed impact. Reflections were iterative and led to emergent processes that included revisiting previously discussed topics, cross-pollination of ideas across panels, and sparking solutions amongst the Team when the panels did not make any recommendations or recommendations were not feasible.

**Conclusions:**

When paired with end-user engagement methods, brief reflections can facilitate systematic examination of end-user input, particularly when the engagement strategy is robust. Reflections offer a forum of accountability for researchers to give careful thought to end-user recommendations and make timely improvements to the study conduct. Reflections can also facilitate evaluation of these recommendations and reveal end-user-driven strategies that can effectively improve study conduct.

**Trial registration:**

ClinicalTrials.gov (NCT04581434) on October 9, 2020; https://clinicaltrials.gov/ct2/show/study/NCT04581434?term=NCT04581434&draw=2&rank=1.

## Background

Engaging end-users in health services and implementation research has become increasingly important to study funders, research teams and end-users. Participatory research methods allow for inclusive and meaningful engagement of end-users, beneficiaries, and other invested parties in research. These diverse arrays of methods are “reflexive, flexible and iterative” [1], and offer opportunities for those outside of the research team to be active contributors to the research process. Contributions can include input on study design, recommendations on the execution of study methods, creative strategies to disseminate findings, and fine-tuning an innovation for implementation into clinical practice. In fact, in implementation science, end-user engagement is embedded in several implementation strategies—e.g., conduct local consensus discussions, use advisory boards and workgroups [2]. Existing literature outlines how to design partner-engaged studies, discusses processes for research partnership development, and offers examples of the types of study-related input partners and end-users can provide [3–5]. Once partnerships have been established, the documentation of end-user input can be achieved through various methods such as qualitative interviews or discussions [6, 7] and can occur after each contact with the end-user or after all end-user engagement has concluded. However, less is known about methods the research team can employ to capture input in real-time and appraise the feasibility and potential value-added of incorporating that input into a study when the engagement strategy is (a) over a long period of time, (b) involves multiple engagement groups and/or (c) requires end-users to provide input about various elements of a research study versus a single element (e.g., development of the content of an intervention).

Reflection is a continuous learning process through which experience and observation are leveraged for future improvement. It has underpinnings in the social sciences—psychology, anthropology, and others. For example, in psychological treatments such as cognitive therapy, patients learn to examine their thoughts around past and current events to gain insight and improve their thinking over time. Mental health therapists can sharpen their therapeutic skills through ongoing, reflective discussions with their clinical supervisor. The application of reflection processes can also be seen outside of the clinical setting in quality improvement (QI) processes and implementation research studies. For example, Plan-Do-Study-Act is a well-established QI strategy that is used to execute changes in healthcare settings [8]. During the Study step, answering key questions such as whether the change worked out as planned and what lessons were learned require the QI team to reflect on the change process. In implementation research, reflection has been used to improve the effectiveness of complex implementation strategies, document the evolution of the implementation of clinical innovations, and document study modifications and their impact through methods like reflective writing and reflection discussions and questionnaires [7, 9–11].

Like QI and implementation research, end-user engagement in research can be iterative and nuanced. Some end-user input, such as feedback on study conduct, can be time-sensitive, requiring a dynamic method like a reflection process to help monitor and incorporate the input provided. To our knowledge, no published studies have applied reflection processes to complement end-user engagement in research. We paired a process of “brief reflections” with our end-user engagement methods as part of a supplemental evaluation of our engagement plan for “The Comparative Effectiveness of Trauma-Focused and Non-Trauma- Focused Treatment Strategies for PTSD among those with Co-Occurring SUD (COMPASS)” study. COMPASS was a two-arm comparative effectiveness pragmatic randomized controlled trial to determine the effectiveness of trauma-focused psychotherapy versus non-trauma-focused psychotherapy for Veterans with co-occurring posttraumatic stress disorder and substance use disorder (PTSD/SUD) who were entering substance use treatment within the Department of Veterans Affairs (VA) [12]. The study included engagement of three critical groups—Veterans, clinicians, and healthcare leadership—to gain input about the study design, procedures, data interpretation and dissemination of findings.

The COMPASS study began approximately two months after the onset of widespread public health measures designed to slow the spread of COVID-19 in the U.S. Due to the pandemic, some aspects of the study conduct changed immediately (e.g., increased virtual delivery of study psychotherapy, elimination of in-person meetings with our panels) and others unfolded throughout the study period. Engagement of our panels was essential to identifying pandemic-related disruptions, exploring options for addressing those disruptions, and monitoring the impact of employed changes on successful implementation of study procedures such as recruitment, assessment, and retention. The brief reflections process was added to the study to serve two purposes—first to systematically review how our engagement groups assisted in adjusting aspects of the study to accommodate COVID-19 restrictions and second, to facilitate evaluation of the impact of those adjustments on study conduct.

In this paper, we first outline our engagement and brief reflections processes. We then describe the integration of multiple data sources used in analyses to close the loop between what was recommended by our engagement groups, how those recommendations were implemented, and their impact on the study conduct. Finally, we present exemplars of end-user driven changes to the COMPASS study for illustration.

## Methods

### Study overview

The COMPASS study protocol details are published elsewhere [12], but briefly, the study occurred at 14 VA medical centers. Each of the study sites was managed by a team of at least one Local Site Investigator (LSI) and at least one Study Coordinator who were responsible for the day-to-day study operations at the site. Each site was tasked with enrolling a total of 30 patients for a study total of 420 patients. Study patients were randomized to receive trauma-focused psychotherapy or non-trauma-focused psychotherapy after enrolling in concurrent treatment-as-usual for substance use disorder. The main outcomes of the study were PTSD severity and PTSD treatment dropout. Assessment occurred prior to treatment initiation, immediately following treatment completion or discontinuation, and again 3- and 6-months following treatment. Assessments included clinical diagnostic interviews which were conducted by independent assessors and self-report surveys. Study treatment was delivered by existing VA therapists embedded in PTSD and SUD clinics. For this paper, we conducted a mixed-methods supplemental evaluation that included brief reflections to identify and understand the impact of end-user recommended-changes on the study conduct.

### End-user engagement

We formed key partnerships during the study proposal development and study execution to maximize our ability to generate trustworthy and valid findings directly relevant to Veterans with PTSD/SUD, PTSD/SUD treatment providers, and healthcare system leaders. Our partnership development aligned with the steps recommended by Israel et al. 2005 – [1] self-reflect on the research team’s capacity, resources, and gaps; 2) identify potential partners; 3) negotiate the research question(s); and 4) create a structure to sustain partnerships [4]. Our COMPASS research team included a range of expertise to successfully execute the study including PTSD, SUD, clinical trial design, treatment fidelity, statistical and qualitative methods, end-user engagement, and implementation of clinical innovations. Our expertise spanned several academic and leadership levels (e.g., Assistant Professors; Associate Director of VA Health Services Research Center). However, self-reflection showed that our research team did not include Veterans and had few clinicians who would implement the therapies being studied or high-level VA and non-VA health care leaders who could use the study findings to inform policies. With this in mind, we sought potential partners who would fill these gaps. During study development, collaboration with VA clinicians helped refine our research questions (e.g., identifying patient characteristics that may differentially affect treatment response). Finally, we formed panels to sustain clinician and other partner engagement throughout the study—a Veteran Consultant Panel (VCP), a Clinician Engagement Panel (CEP), and a Study Advisory Committee (SAC). We used various channels to recruit members for our engagement panels. Once established, all panels had regular meetings that were held quarterly and by video conference due to the COVID-19 pandemic. Meetings were facilitated by a trained staff facilitator and a study co-investigator and 2–3 members of the COMPASS research team for the VCP, and the study PI and Co-PI for the CEP and SAC.

#### Veteran consultant panel (VCP)

We recruited potential members of the VCP through provider referrals from each of the COMPASS study sites. Our goal was to include Veterans with lived experience with PTSD and SUD. Candidates were interviewed to assess their interest in the project, whether they had experienced PTSD and struggled with substance use in the past and if they could commit to participating in quarterly video meetings and eventually travel to Minneapolis for an in-person meeting. From these interviews, we selected 12 Veterans who were diverse in age/service era, gender, race, and geographic location to join the panel. Panelists were provided a position description and signed a confidentiality and membership agreement to ensure all agreed with the ground rules of the group and maintained one another’s confidentiality. Before each 2-hour meeting, panelists were sent a packet of materials to review.

#### Clinician engagement panel (CEP)

For the CEP, we wanted therapists who could provide their perspectives and experiences on providing PTSD therapy to patients early on in their treatment for comorbid SUD. Eight COMPASS study clinicians were recruited for the CEP through site PI referral and group emails to study clinicians at each study site. All interested clinicians were included on the panel. During the 50-90-minute meetings, panelists were given study progress updates and asked to provide updates on study progress at their sites. There was then a group discussion on a pressing study issue.

#### Study advisory committee (SAC)

The study principal investigator (PI) and Co-PI identified and approached members for the SAC based on their relevant expertise and leadership roles. Members included 11 research and clinical leaders from both VA and non-VA health organizations. The SAC met for 1 h to hear about study progress and advised the study leaders on ways to enhance the study procedures and impact.

### Brief reflection process

The main goal of the brief reflections was to systematically translate recommendations made by the engagement panels into actionable steps to improve study conduct. Reflections were a venue for the study team to carefully consider all recommendations. Specifically, reflections were semi-structured discussions with members of the COMPASS team who facilitated and attended the engagement panel meetings [hereafter referred to as “COMPASS Team” or “Team”]. Reflections were facilitated by a co-investigator of the COMPASS study who was familiar with the components of the study but who was not directly involved in conducting the engagement panel meetings so that questions could be asked objectively.

Reflections occurred via video conference one-to-three weeks after each panel meeting to minimize recall bias and capture the COMPASS Team’s decision-making early in the process. There were 16 brief reflections—5 for the VCP; 6 for the CEP; and 5 for the SAC. Using a semi-structured guide, the reflections inquired about key recommendations that arose from the panel meetings, if and how the COMPASS Team planned to implement recommendations, the potential relationship between recommendations and/or implementation plans to the COVID-19 pandemic, and how best to evaluate the impact of implementing the recommendations on the study. Additional probes were used as needed to clarify and follow up on responses given by the Team, or to inquire about the relatedness of a present topic to a topic that was discussed in a prior reflection. All reflections were recorded and professionally transcribed.

### Analysis

We executed a mixed-methods supplemental evaluation that merged the reflections with other data sources from the study to assess the impact of study changes. Reflections were analyzed using a rapid-analysis approach using summary sheets and matrices that were organized by our domains of interest (e.g., relationship of the topic to the pandemic; recommendations made by the panel; plan to implement recommendation; etc.) for each topic discussed at each panel meeting. The analysis team reviewed two reflections together to calibrate summaries. The remaining brief reflections were assigned across the analysis team. Meetings were used to discuss questions and reach consensus. Once all summaries were complete, they were combined into a single summary matrix to allow for synthesis of the findings.

The reflections identified changes recommended by our engagement panels, the COMPASS Team’s initial thoughts about whether to implement these changes and possible ways to assess their impact on the study. To identify *actual* changes made and evaluate *actual* impact, the brief reflections facilitator walked through the summary matrix with the COMPASS Team to identify which recommended changes were made and what information was collected through the different components of the study that could be used to evaluate the impact of the changes. To assess the impact of each executed recommendation on the study, we consulted other study data sources. Example sources described below correspond to the exemplar recommendations presented in the Results.

#### Examples of other study data sources

- *Site Reports*. Each LSI received a report two times a month about the number of participants screened, consented, assessed, and randomized. The reports helped LSIs to monitor the extent to which they met their recruitment goals. Later in the study when participants began to complete study treatment, the reports also included the number and proportion of participants who had completed their immediate posttreatment and 3- and 6-month follow-up assessments and surveys to monitor study retention.
- *In-Session Assessment Completion Report*. For each COMPASS therapy session, participants were asked to complete a PTSD Checklist-5 (a measure of PTSD symptoms) and a Brief Addiction Monitor (a substance use progress-monitoring measure). For each measure, we calculated the proportion completed per quarter of the study—both overall and for each of the 14 study sites.
- *Therapist Burnout Survey*. Following reports of therapist burnout, the Co-PI led the Team in developing a survey to assess level of burnout among the COMPASS study therapists. Therapists were emailed the 5-item survey that asked about the degree to which the study contributed to their burnout and the value of strategies (e.g., virtual educational opportunity) that could improve their experience as a study therapist [very valuable to not at all valuable].

## Results

The three engagement panels recommended changes to the study in a multitude of areas. We synthesized these into eight categories: *enhancing recruitment; study assessment completion; creating uniformity across Study Coordinators; building Study Coordinator connection to Veteran participants; mismatch between study procedures and clinical practice; therapist skill with patients with active substance use; therapist burnout; and dissemination of study findings.* Table 1 provides details about each category, study-related issue, sample panel-driven change that was implemented, and the impact of the change on the study. We found that in each of the categories, there were multiple changes that our panels recommended, and, in many cases, the Team implemented more than one change. Below, we provide examples of changes recommended by the panel that directly impacted the study conduct and examples of changes that led to mixed impact. We also provide examples of three processes that emerged as reflections were conducted–revisiting topics, cross-pollination, and sparked ideas.

**Table 1:** Summary of categories of panel-recommended changes

Categories (Associated Engagement Panel)	Details of the Study Issue	Sample Recommendation Implemented	Impact on Study
**Enhancing recruitment** (VCP, CEP, SAC)	Recruitment had slowed overall; Variability in recruitment success across study sites	Look at granular data (e.g., conversion rate from screened to randomized)	Recruitment rates improved but study extension required
**Study assessment completion** (VCP, SAC)	Participants were no-showing baseline assessments; Participants were not completing 3- and 6-month posttreatment assessments	Increase attempts to reach participants	Retention rates went from 59% in May 2022 to 74% in December 2023
**Creating uniformity across Study Coordinators** (CEP)	Coordinators were not supporting study therapists at the same level across study sites (e.g., helping to manage therapy-related paperwork)	Create “Best Practices” document of Coordinator duties and share with all Coordinators	Therapists reported an improvement in Coordinator support received
**Building Study Coordinator connection to Veteran participants** (VCP)	Concern among VCP that Coordinators are not Veterans and therefore will have difficulty connecting with Veteran participants	Invite Coordinators to VCP meeting*	Coordinators more prepared to interact with Veteran study participants
**Mismatch between study procedures and clinical practice** (CEP)	Clinic procedures did not always align with study procedures (e.g., length of time patient’s timeslot remains available after no-shows)	Give therapists flexibility in length of time timeslot is held for study participants	Study more aligned with clinical practice; Unknown impact on study
**Therapist burnout** (CEP, SAC)	Therapists experienced burnout during the pandemic	Send therapists and their supervisors a letter recognizing their work on study	13 out of 14 sites continued remained a study site after the study extension
**Therapist skill with patients with active substance use** (CEP)	Study therapists were experts in treating PTSD and had less experience working with patients with SUD	Use therapist-requested educational seminars to enhance therapist skills with patients with SUD*	Unknown—study in data analysis phase
**Dissemination of study findings** (VCP, CEP, SAC)	n/a	Use non-VA channels	Unknown—study in data analysis phase

*In rare instances, no specific recommendations were made by our panels; however, the COMPASS Team generated ideas for change during brief reflection.

### Examples when panel-recommended changes impacted study conduct

#### Study assessment completion

In mid-to-late-2021, the COMPASS Team noticed there were no-shows/rescheduled appointments for the baseline assessment (e.g., 25% in August and September 2021), and wondered if one reason was the sensitivity of questions being asked over the phone (e.g., asking about details of traumatic experiences), and whether this was compounded by the inability to recruit patients in person. The original recruitment plan included in-person recruitment from SUD or PTSD clinics so that trust could be established with the Study Coordinator prior to subsequent contacts which would occur by phone. Due to the pandemic, all contact shifted to the phone.

VCP members were queried about strategies that could boost baseline assessment completion. VCP members were specifically asked about the sensitive nature of discussing trauma experience over the phone. VCP members suggested that Coordinators not “tiptoe” around issues as Veterans have experience talking about sensitive topics. At the same time, listen and prepare the Veteran as the call progresses. One VCP member stated, “We have mostly likely been asked these questions before. Let the veteran know that it is very basic but prepare them that [the trauma] will be mentioned.” Another shared that, “I tend to be an over-sharer. I lost my voice working in the [military], so I fought to get it back. Have the coordinator let that happen. The coordinator can say, ‘thank you for sharing that. Is it okay that we transition to the next question?’” As a result of the feedback received, the Team enacted the following changes: (1) provided additional training to the Study Coordinators to emphasize certain information during contact with study participants (e.g., how the study obtained their names) and ways to let the participant drive discussions about trauma experience to establish trust; and (2) encouraged referring clinicians to mention to their patient that a Study Coordinator will call them. No-shows persisted and SAC members were queried about the issue in November 2021. They suggested the Team provide a bonus incentive to those who completed baseline interview on time (i.e., did not no-show or reschedule) which the Team reflected was “a fantastic idea…something to really, really consider” based on the study budget. The Team assessed the budget and started distributing bonus checks in December 2021. Through April 2023, 482 checks were distributed, increasing from 13 to 64% of assessments in that timeframe and suggesting a decrease in no-show rates for the baseline assessment.

#### Mismatch between study procedures and clinical practice

The study protocol specified patients would complete a PTSD Checklist-5 (PCL-5) and a Brief Addiction Monitor (BAM) at each therapy session. However, Study Coordinators notified the Team that therapists were unclear on whether the collection of these measures were their responsibility and measures were not consistently administered. Specifically, half of the study sites (*n* = 7) were missing the PCL-5 and/or BAM for ≥ 10% of sessions. The issue was discussed with the CEP. During the brief reflection, the Team noted that this may in part be due to the impact of shifting to virtual delivery of care as part of the COVID-19 restrictions:

The reason why this has become an issue is because of the virtual care delivery, and how much harder it is to get those weekly measures that are part of therapy when care is being done virtually rather than in person. Most clinics have procedures that when you check-in, the [clinic staff] gives you your PCL for the week, and then you bring that finished to your therapy session. The therapist doesn’t manage it, and it doesn’t eat up therapy time. And now we’re asking therapists to manage it and it is more likely to eat up therapy time.

CEP members suggested that study-related paperwork be combined into one sheet to allow therapists to better track in-session documents for which they are responsible. The Team believed this would be helpful because therapists were “looking at sheets and trying to juggle electronic versions instead of paper versions [they would normally have] in front of them in the office.” The COMPASS PI also reiterated to each study site that the therapists were responsible for administering the measures. Six months after the problem was identified, the proportion of missing measures started to decline and by the end of the study, only two study sites were missing the PCL-5 and/or BAM for ≥ 10% of sessions.

### Examples when panel-recommended changes had mixed impact

#### Enhancing recruitment

In the early months of recruitment which began in December 2020, there was variability across the 14 sites. At the time, the site reports showed that two sites had not screened any patients for study eligibility and others were not consistently meeting the target of 2 patients/month. Although the COMPASS Team expected some variability, in May 2021, they sought the input of the SAC to identify strategies to reduce the variability. The SAC members made several recommendations—1) the Team could look at more granular data (e.g., conversion rate from screened to randomized; proportion of assessments that occur the day on which they were scheduled) to help tailor strategies for increasing recruitment; 2) in addition to the individualized twice-monthly site reports each site received, data for all sites could be shared to foster friendly recruitment competition and sites with recruitment success could be recognized; and 3) pair Coordinators from higher and lower recruiting sites so they could share recruitment tips.

During the brief reflection, the Team reported they were considering instituting some variation of the recommended changes. For example, regarding the 2nd recommendation, the Team discussed recognition options for high-recruiting sites, but it was unclear if those would also help the low-recruiting sites. When asked, the Team responded, “It varies by site. Sites that are really struggling may need more support and strategies to make things better. For other sites, a little bit of friendly competition might help.” Further, the Team acknowledged that the SAC discussion helped them to consider ways to bolster the high-recruiting sites when the tendency is to focus on low-recruiting sites: “We thought it was a really useful suggestion to not focus all your time and attention on the [low-recruiting sites] and instead [turn some focus] on those you could move from good to great or great to excellent.”

Based on the SAC feedback and brief reflection discussion, the Team instituted the following changes—1) shared recruitment success stories and discussed recruitment strategies that were successful or not yielding progress at the Study Coordinator meetings, 2) shared cross-site data at Local Site Investigator meetings, and 3) top-recruiting sites were recognized during those meetings (e.g., verbal shoutout). While there was some site-specific improvement in recruitment (e.g., one of the two sites that had not screened any patients screened 9 patients/month for the 2nd half of 2021), site reports showed that the overall recruitment had not improved enough to reach the target sample of 420 by the end of the study. As a result, the study PI and Co-PI applied for a study extension with the funder to help reach the target sample, which was awarded in June 2022 and helped to bring the final study sample to 426.

#### Therapist burnout

In multiple brief reflections, burnout among the study therapists was discussed as an ongoing issue. As the Team shared, “the issues of continued therapist squeeze continues…everyone is overworked and [don’t] have enough staff and [feel] tired.” The Team reflected that therapists were trying to balance steadily increasing clinical duties with study responsibilities. They believed the pandemic had led to multiple shifts in modes of treatment delivery (in-person and/or virtual care) and made it more challenging to balance work-life demands.

The Team sought input from both the CEP and SAC members over the course of the study on ways to improve therapists’ study experience as a way to alleviate some of the feelings of burnout. One of the initial recommendations made by the CEP was to distribute letters or certificates to study therapists to recognize their study efforts. The Team created letters of recognition for study therapists and sent copies to therapists’ supervisors and other local site leadership in time for annual performance evaluations. The feedback was very positive: “[Therapists said] that the letters were highly appreciated and just that little positive thing in their day was really impactful.”

However, reports of burnout continued. In addition, to sending another round of letters the following year by request from the CEP, the Team also queried the SAC members about additional strategies. During the brief reflection, the Team shared that the SAC recommended the Team go beyond the CEP and “find out [if] this is something across sites, is it more prevalent at some [sites] or the other…some sort of way of connecting with the therapists and finding out where they’re at, a little brief survey or something.” The Team also considered “drop-in options” where any study therapist can meet with the Team and discuss challenges of being a study therapist during COVID and ways the Team could be helpful. However, this would add “another thing to fit in your schedule.”

The Team created and administered a Therapist Burnout Survey to all 64 COMPASS therapists to assess level of burnout and potential remedies. Thirty-three study therapists completed the survey. About 73% (*n* = 24) reported that the study contributed to their current level of burnout. The most endorsed strategy for making their role as a study therapist more rewarding was virtual educational opportunities (∼ 82%) followed by having virtual office hours about study-related issues (∼ 76%), having an in-person visit from the study PI (∼ 73%), and serving as a champion for concurrent PTSD/SUD therapy at their facility/VA region (∼ 55%).

Based on level of endorsement and feasibility, the Team decided that they would provide educational seminars. To get ideas about seminar content, the Team brainstormed topics at the CEP meeting that followed the survey. The final topics selected were treating patients with active substance use, advanced training in Present-Centered Therapy (the non-trauma-focused therapy arm of the study), and how to reduce treatment dropout. The seminars were presented virtually by COMPASS Team members with related expertise and recorded for therapists’ future reference. No follow-up survey was administered to assess the impact of these changes on burnout. However, there was some evidence that burnout did not fully obstruct therapists’ study participation—13 out of the 14 sites agreed to remain a study site when the study was awarded the extension to reach its recruitment target.

### Emergent processes

In addition to our planned brief reflections, three emergent processes were observed as the reflections were executed. First, there were instances where the Team recognized the need to revisit a topic with the panel that made the initial recommendations to address that topic. This was particularly true when the topic was ongoing versus a one-time issue and/or complex in nature. One topic that was revisited with the panels was therapist burnout. As mentioned above, burnout amongst the study therapists was ongoing given that the study took place during the pandemic. To ensure that the study experience was rewarding, after the Therapist Burnout Survey was administered, the Team followed up with the CEP to discuss which topics would be the focus of the educational seminars requested by the study therapists.

Second, there was cross-pollination of topics/recommendations across the panels that was facilitated by the brief reflections. Cross-pollination refers to times the Team brought a topic/recommendation that arose in one panel to another panel for further feedback. This occurred when the Team thought that the experience or expertise of the other panel would help strengthen the recommended change from the first panel. An example of cross-pollination occurred when study participants were not completing their immediate posttreatment, and 3- and 6-month follow-up assessments. As a result of a discussion with the SAC, the Team decided to survey the Study Coordinators to assess retention strategies being used. The Team also queried the VCP about their perceptions of specific retention strategies (e.g., calling Veteran participants multiple times): “We were concerned that this schedule of multiple calls and multiple ways of contacting [study participants] could be seen as harassing or annoying and [the VCP] did not perceive that at all.” The Team reported that VCP shared that “’persistence is caring’” and the multiple calls “meant that someone cared about them and was looking out for them.” After enacting the various strategies including multiple calls, the site reports showed that across the sites, retention rates went from 59 to 74% between May 2022 and December 2023.

Finally, in rare instances, no specific study-change recommendations were made by the engagement panels or the panel recommendations were not feasible. However, the brief reflection discussion sparked ideas for changes among the COMPASS Team based on panel-member reactions to a study issue. For example, during a VCP meeting where the Team shared that recruitment was below 80% of the target sample, the Team reported that VCP members believed that one reason might be “that no one really knows what it’s like to be a Veteran unless you’re a Veteran,” and thus potential participants may not open up to Study Coordinators who were not Veterans. Rather, the VCP believed it would be more effective if Veterans recruited for the study. Since the COMPASS Team did not include Veterans, the Team discussed the idea of having the Study Coordinators join a VCP meeting to interact with VCP members, as they had done in the past, as a way “to grow the sensitivity of [Team members] who were not Veterans.” The Team believed this was a viable recommendation because the Study Coordinators’ presence was well-received by both parties in a past VCP meeting: “We had the one meeting where [they] were both there together and [the Coordinators] tried to share some of [their] experiences and [the VCP] really appreciated it, the coordinators felt like they gained some sensitivity.”

## Discussion

Our brief reflections process during the COMPASS study was used to systematically examine recommendations made by our engagement panels to improve study conduct. Although the process was initially incorporated into the study to help monitor changes resulting from COVID-19 restrictions, brief reflections were a medium through which the Team could process *any* study-related topics/issues and the recommendations made by the panels. During the reflections, the Team carefully considered to the feasibility of implementing recommendations within a large healthcare system, given regulatory requirements, finite research resources, and the additional restrictions imposed during the COVID-19 pandemic. Our process of brief reflections also facilitated consideration for ways to evaluate the impact of those changes in near real-time (e.g., study site reports).

Reflections revealed that incorporating engagement panel recommendations was a process that was iterative and non-linear. Cross-pollination and revisiting topics with our engagement groups facilitated richer engagement and execution of recommendations around study issues. In addition, panel engagement and the reflection discussions sometimes sparked ideas among the COMPASS Team to address study-related issues when the panels did not make specific recommendations or recommendations could not be integrated feasibly.

Though it has long-standing utility in the social sciences, using reflection as a research methodology is relatively new. Similar methods have been used in a handful of implementation studies—for example, reflective writing with implementation facilitators or using reflections to document adaptations to implementation studies [7, 9–11]. Its application to end-user engagement and effectiveness trials such as the COMPASS study is unique and advances the literature in participatory research methods. Namely, our study showed that brief reflections is an effective complement to end-user engagement by helping the research team to be targeted and thoughtful in implementing panel-recommended changes. It also allowed the Team to recognize early when panel-proposed solutions were not going to drastically improve a study issue and to pivot accordingly. For example, when recruitment slowed, the Team implemented several panel-recommended changes. Despite some improvement, recruitment targets were still not consistently met across the study sites. As a result, the Team applied for and was awarded an extension to reach the target sample. Further, the brief reflections provided an additional level of accountability for the Team such that the process of seeking feedback from our engagement panels was not just performative, but panel feedback was well-thought-out and implemented if feasible.

Pairing reflections with other study data sources to evaluate recommendation impact is a unique strategy. The brief reflections facilitated evaluation of the impact of panel-recommended changes. Evaluations of end-user engagement tend to focus on real-time surveys that provide nominal information, qualitative interviews conducted after the study, or post-hoc, non-standardized personal reflections by researchers [13, 14]. Analysis of our brief reflections and other COMPASS study data sources showed that overall, the engagement panels contributed to and strengthened the COMPASS study design and conduct. Across the eight categories, there were specific strategies that were panel-recommended. Some panel-recommended changes effectively addressed issues that arose (e.g., low rates of posttreatment assessment completion). Others had mixed impact (e.g., providing therapists with recognition letters may have contributed to a positive study experience but did not alleviate burnout). Perhaps this was because complex issues such as burnout require multiple strategies and attempts.

Our evaluation had some limitations. Our strategy for brief reflections and closing the loop with additional data sources was largely effective in identifying how engagement panel recommendations were implemented. However, in some cases, it was challenging to determine which recommendations influenced the quality of the research study because multiple changes were implemented to address the concern. Thus, even though the study issue was resolved, it was not always possible to pinpoint which recommended change or strategy led to specific improvements in study conduct. An initial panel recommendation led to a cascade of activities, such as greater awareness of related issues among the Team, additional information gathering, and webs of changes related to the new information and awareness. This cascade of influence is difficult to capture, and any given change implemented is difficult to unambiguously tie back to the original recommendation. For example, to assess and address therapist burnout, the Team surveyed all study therapists as recommended by the study advisory group. While this initial survey led to concrete changes to improve therapists’ study experience (e.g., providing educational seminars), it is debatable if it is reasonable to link the benefits from the seminars to the original recommendation (a survey). Yet, without the original recommendation, the cascade of activities would have never happened.

Future research could consider building in assessment of study staff satisfaction with study procedures upfront. Trends in data before and after implementing recommended changes may provide a signal as to how recommended changes influence staff experience. Despite these limitations, our brief reflections process facilitated real-time action and evaluation of study conduct and progress. Together, we learned that our engagement panels have provided valuable recommendations to make timely improvements to the study. In addition, the evaluation highlighted strategies that are more likely to improve how a study is conducted.

## Conclusions

Participatory research methods are increasingly incorporated into research trials. When paired with end-user engagement methods, brief reflections are a feasible process that can facilitate systematic examination of end-user input, particularly when there is a robust engagement strategy that includes gathering input from multiple types of end-users, on various elements of a research study, and over a long period of time. They also provide a forum of accountability for the research team to appraise end-user recommendations and make timely improvements to the study conduct. In addition, reflections can facilitate evaluation of end-user engagement and identify strategies for effectively improving study conduct.

## Data Availability

The datasets generated and/or analyzed during the current study are not publicly available due the need to protect individual privacy among engagement panel members and study team members participating in the method described.

## Abbreviations

CEP: Clinician Engagement Panel
COMPASS: Comparative Effectiveness of Trauma-Focused and Non-Trauma- Focused Treatment Strategies for PTSD among those with Co-Occurring SUD
LSI: Local Site Investigator
PI: Principal investigator
PTSD/SUD: Co-occurring posttraumatic stress disorder and substance use disorder
QI: Quality improvement
SAC: Study Advisory Committee
VA: Department of Veterans Affairs
VCP: Veteran Consultant Panel
